# Human microbiota modulation via QseC sensor kinase mediated in the *Escherichia coli* O104:H4 outbreak strain infection in microbiome model

**DOI:** 10.1186/s12866-021-02220-3

**Published:** 2021-06-02

**Authors:** Tamara Renata Machado Ribeiro, Mateus Kawata Salgaço, Maria Angela Tallarico Adorno, Miriam Aparecida da Silva, Roxane Maria Fontes Piazza, Katia Sivieri, Cristiano Gallina Moreira

**Affiliations:** 1 Department of Biological Sciences, School of Pharmaceutical Sciences, São Paulo State University (UNESP), Araraquara, SP Brazil; 2 Department of Food and Nutrition, School of Pharmaceutical Sciences, São Paulo State University (UNESP), Araraquara, SP Brazil; 3 Department of Hydraulics and Sanitation, School of Engineering of São Carlos, University of São Paulo (USP), São Carlos, SP Brazil; 4 Bacteriology Laboratoty, Butantan Institute, São Paulo, SP Brazil

**Keywords:** EAEC, QseC, Microbiota, SHIME®

## Abstract

**Background:**

The intestinal microbiota plays a crucial role in human health, adjusting its composition and the microbial metabolites protects the gut against invading microorganisms. Enteroaggregative *E. coli* (EAEC) is an important diarrheagenic pathogen, which may cause acute or persistent diarrhea (≥14 days). The outbreak strain has the potent Shiga toxin, forms a dense biofilm and communicate via QseBC two-component system regulating the expression of many important virulence factors.

**Results:**

Herein, we investigated the QseC histidine sensor kinase role in the microbiota shift during O104:H4 C227–11 infection in the colonic model SHIME® (Simulator of the Human Intestinal Microbial Ecosystem) and in vivo mice model. The microbiota imbalance caused by C227–11 infection affected ỿ-Proteobacteria and *Lactobacillus* spp. predominance, with direct alteration in intestinal metabolites driven by microbiota change, such as Short-chain fatty acids (SCFA). However, in the absence of QseC sensor kinase, the microbiota recovery was delayed on day 3 p.i., with change in the intestinal production of SCFA, like an increase in acetate production. The higher predominance of *Lactobacillus* spp. in the microbiota and significant augmented *qseC* gene expression levels were also observed during C227–11 mice infection upon intestinal depletion. Novel insights during pathogenic bacteria infection with the intestinal microbiota were observed. The QseC kinase sensor seems to have a role in the microbiota shift during the infectious process by Shiga toxin-producing EAEC C227–11.

**Conclusions:**

The QseC role in C227–11 infection helps to unravel the intestine microbiota modulation and its metabolites during SHIME® and in vivo models, besides they contribute to elucidate bacterial intestinal pathogenesis and the microbiota relationships.

**Supplementary Information:**

The online version contains supplementary material available at 10.1186/s12866-021-02220-3.

## Background

The human digestive tract hosts hundreds of microorganism’s species that are collectively known as microbiota. The gut microbiota is a complex and dynamic ecosystem that includes bacteria, archaea, virus, fungi and protozoa [1]. The colon albeits the largest and most diverse microbial population in the human intestine, with approximately 10^12^ bacterial cells per gram of luminal contents [2]. This community is predominantly composed of Firmicutes and Bacteroidetes phyla, followed by Protobacteria and Actinobacteria [3]. The intestinal microbiota composition may influence the functions of the cardiovascular, nervous and endocrine systems [4–6]. Moreover, the microbiota provides many benefits for human health such as the development of the digestive and immune systems, production of vitamins, metabolization and availability of nutrients, as well as protection against several pathogens [7, 8]. The balance between commensal and potentially pathogenic bacteria is a central element of human health. Thus, the microbiota dysbiosis may result in greater susceptibility to the development of infectious and chronic diseases [9, 10].

Short-chain fatty acids (SCFA) are the main metabolites from polysaccharide fermentation by anaerobic bacteria in the colon, SCFA such as acetate, propionate and butyrate represent about 90–95% of their composition [11, 12]. SCFA play an important role in maintaining gut health, such as protection, energy source and physiological homeostasis [13]. Acetate is the main SCFA produced in the colon, and also the most abundant in the bloodstream [14, 15]. The acetate activity in the colon has an anti-inflammatory role, it helps the pH balance, increases blood flow and improve oxygen uptake; it is also used as a substrate for the butyrate production by other members of the microbiota during the cross-feeding process [16].

Infectious diarrhea is a major global public health problem, unfortunately it ranks high in mortality rate among all ages, and amongst infants under 5 years old is classified within the top 5 mortality cause [17]. Enteroaggregative *E. coli* (EAEC) is an important etiologic agent of acute and persistent diarrhea (≥14 days) for both children and adults worldwide [17, 18]. This pathogen is known to produce a thick biofilm and a typical adhesion pattern in cell cultures similar to stacked bricks, mediated mainly by aggregate adhesion fimbriae (AAF). Five variants of this fimbria have been described (AAF/I-V), all of them encoded by pAA plasmid and dependent of the AggR transcriptional regulator, as an activator. AggR has been described in EAEC as an important transcriptional activator of at least 44 virulence genes, such as those encoding AAFs and a dispersin (antiaggregation protein) [19–24]. Nonetheless, this pathotype is a genetically very heterogeneous bacterial group, whereas recent studies have found virulence factors statistically correlated with disease, although EAEC complete pathogenesis process remain unclear [25, 26].

In 2011, a large outbreak of foodborne bloody diarrhea began in Germany and quickly spread to other countries, resulting in 3816 sick people and 54 deaths [27]. The O104:H4 strains were immediately sequenced and directly linked to these cases. Interesting most of these strains were Shiga toxin (Stx) type 2 (Stx2) producers, usually found in enterohemorrhagic *E. coli* (EHEC) and other Shiga toxin-producing *E. coli* (STEC), utmost related to hemorrhagic colitis (HC) and hemolytic uremic syndrome (HUS) cases. The outbreak strains isolated showed a deadly combination of EAEC virulence factors and Stx2 in these highly virulent strains [28]. The O104:H4 strain C227–11 is the main representative isolate from this outbreak in Europe [29]. Additionally, this strain produces SPATEs (Serine Protease Autotransporters of Enterobacteriaceae) [30, 31], such as Pic, SigA and SepA involved in the infectious niche establishment [29, 30]. Moreover, this C227–11 outbreak strain encodes AAF/I (aggregative adherence fimbriae I), the antiaggregation dispersin, and other important adhesins related to biofilm formation, such as LPF (long polar fimbriae) and Iha (IrgA homologue adhesin) [28, 32].

The coordination of metabolic and pathogenic mechanisms in bacteria are mediated by chemical signaling via 2-component systems, also known as *quorum sensing* [33]. The QseBC 2-component system, first described in EHEC [34], plays a crucial role in regulating the virulence genes expression of important human enteropathogens [35]. This system is composed by inner membrane histidine kinase sensor QseC and a cytoplasmic response regulator QseB. QseC mediates inter-kingdom signaling by detecting host stress hormones, epinephrine and norepinephrine, in addition to Autoinducer-3, a molecule produced by a diversity of Gram-positive and negative bacteria [34, 36, 37]. In EHEC, QseC detects these environmental signals, and activates the virulence genes transcription of LEE pathogenicity island, motility and Shiga toxin [38]. In addition, QseC homologues are found in at least 25 bacterial pathogens [39]. Recently, its role has been described in the EAEC pathogenicity, whereas the QseC sensor kinase was shown to be important during in vitro and in infection via Type I fimbriae adherence [40–42].

There are limitations to the use of animal models to investigate EAEC infection, since the pathogen has multiple virulence features and the models only mimic partially the pathogenesis without diarrhea, as well as ethical limits to conduct clinical trials [43]. Animal models in EAEC such as oral mice infection is employed to mimic the natural oral route, with antibiotic depletion to favour EAEC colonization [44], this model seems very appropriated to compare the intestinal murine colonization in vivo with a reactor in vitro model colonized by human microbiota. Therefore, a colonic model, such as the Simulator of the Human Intestinal Microbial Ecosystem (SHIME®) [45, 46] becomes an excellent tool to correlate the human microbiota and bacterial pathogenesis. This system mimics physiological conditions, such as pH, temperature, transit time, enzymatic digestive activity, and gut microbiota in the gastrointestinal tract [47]. Vast amount and variety of metabolites have been described by gut microbiota, the SCFA are abundant and important energy sources for intestinal colonic cells and microbial population, besides an important role in the host metabolism and immune system homeostasis [48].

The present study has investigated the QseC role in the microbiota shift and metabolites composition in the intestinal microbiome during O104:H4 Stx + outbreak strain infection in the human colonic model SHIME® and mice infection.

## Results

### QseC sensor kinase modulates human intestinal microbiota shift during C227–11 Stx + infection during SHIME® infection

**Fig. 1:**
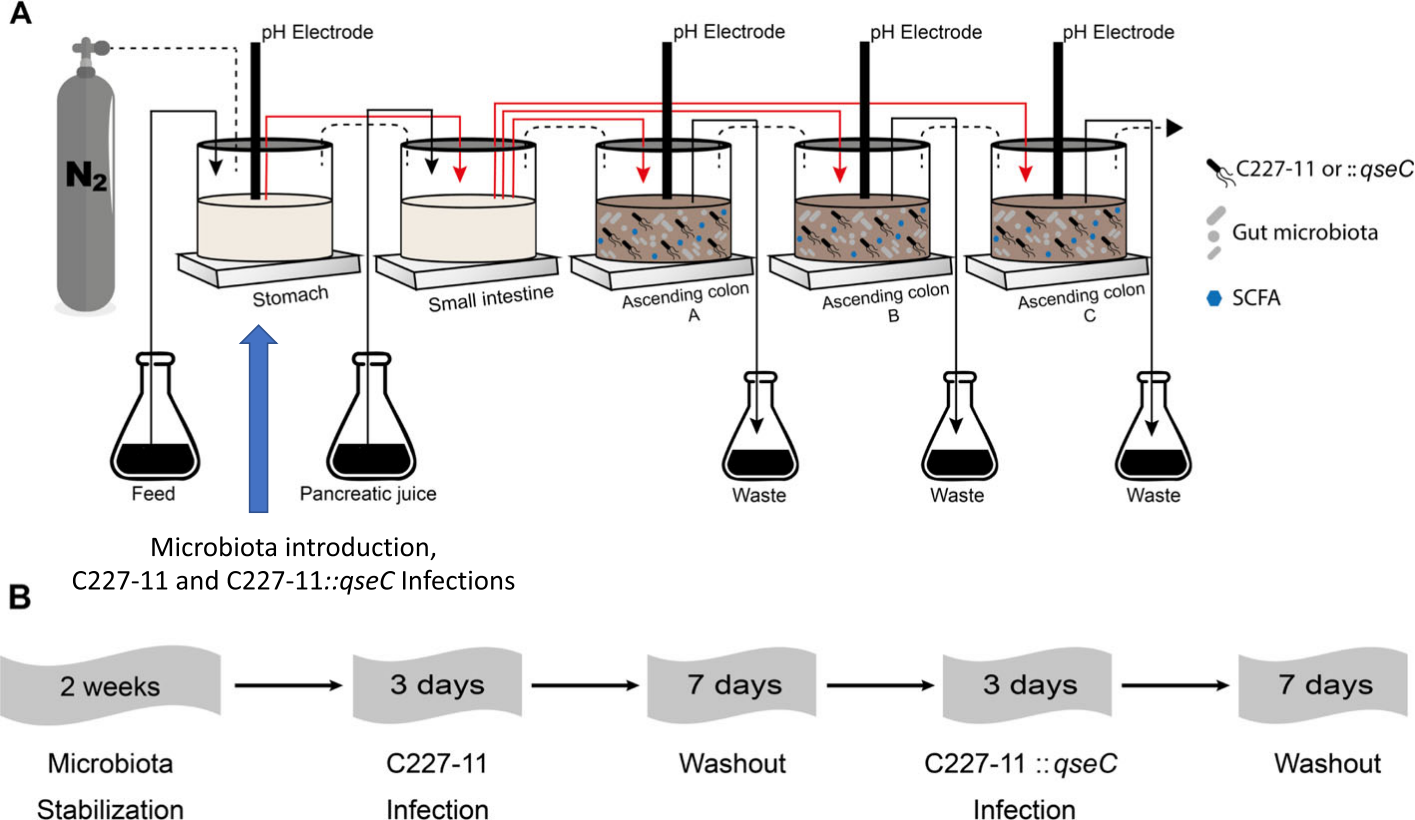
SHIME® infection model overview employed with all controlled containers representing the gastrointestinal tract, with stomach and its feed, small intestine and its pancreatic juice and the ascending colon triplicates employed here. The arrows indicate the flow direction of the pumps, dashed lines for gas and solid lines for liquids (**a**). Experimental protocol with all period steps developed in 5 weeks during C227–11 infection (**b**). C227–11 and C227–11::*qseC* strains presence in output was verified by PCR amplification

**Fig. 2:**
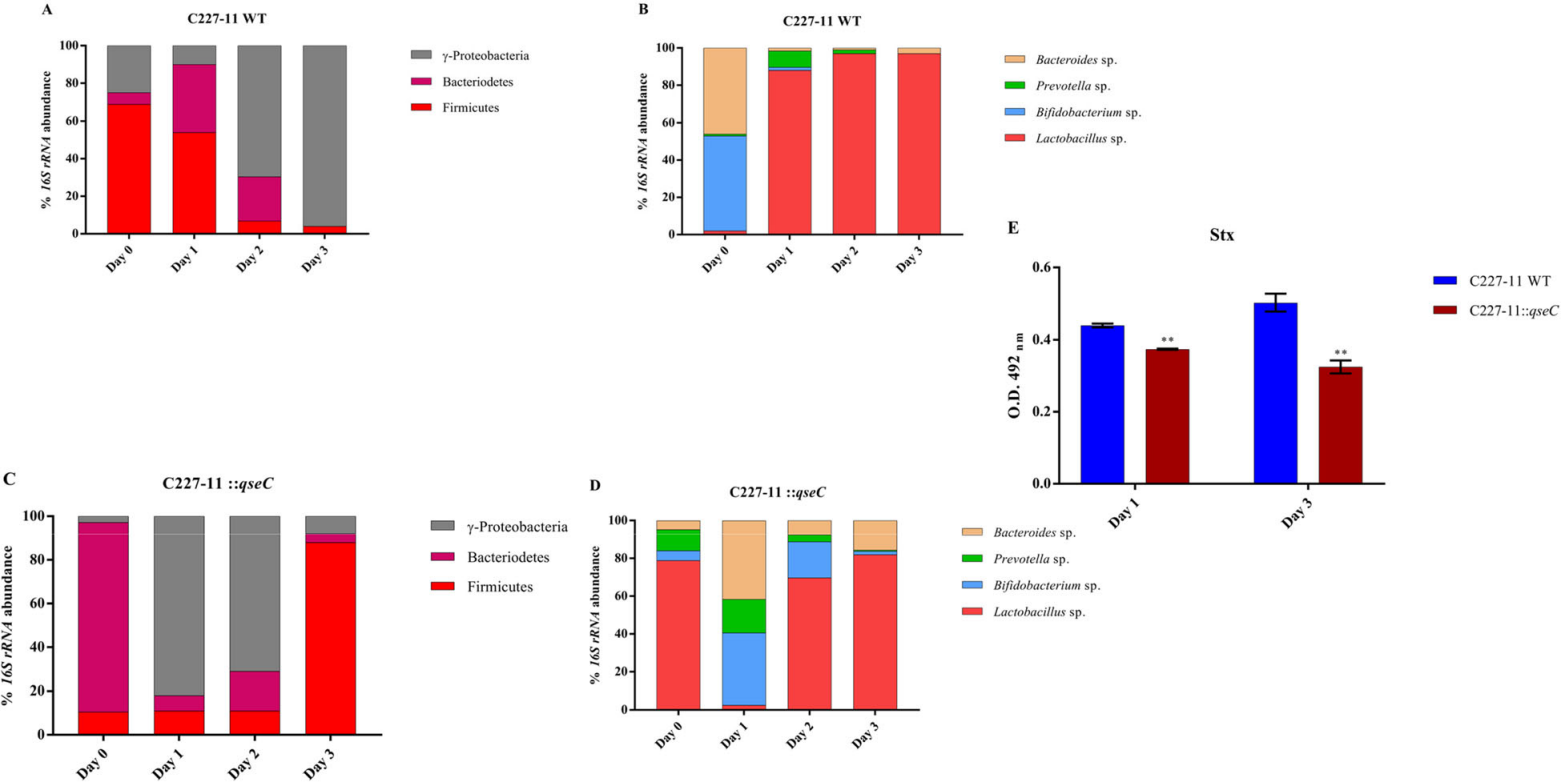
Microbiota predominance modulated via QseC during C227–11 infection in the SHIME® model. Relative microbiota abundance analysis via qRT-PCR of 16 *s* *rRNA* of phyla and genera. Microbiota composition from days 0 to 3 p.i with strain C227–11 infection, respectively, phyla and genera (**a** and **b**), and with strain C227–11::*qseC* infection, respectively, phyla and genera (**c** and **d**). ELISA Immunoassay capture to measure the Stx levels from the output collected during the SHIME® infection, day 1, ** *p* = 0.002 and 3 p.i., ** *p* = 0.009 (**e**). The statistical significance analyzes were performed on GraphPad Prism 7 via t-test

The QseC sensor kinase role in intestinal microbiota composition was verified during the Shiga toxin producing O104:H4 *E.coli* infection. The C227–11 and C227–11*::qseC* strain were employed during in vitro infection of controlled colonic SHIME® model, to mimic the human gastrointestinal tract, evaluating the intestinal microbiota abundance through C227–11 infection [49], as detailed illustrated (Fig. 1a and b). Here, we have analyzed the Firmicutes, Bacteroidetes and ỿ-Proteobacteria phyla presence measuring their levels in the system, as well as the abundance of bacterial genera present in the human intestine as *Lactobacillus* spp*.*, *Bifidobacterium* spp., *Prevotella* spp., and *Bacteroides* spp. [3, 50]. The viable bacterial cells in the system were measured via qRT-PCR from day 0 to day 3 after C227–11 and C227–11*::qseC* strains infection. Initially, there was a 30% increase in Bacteroidetes and a 15% decrease in both ỿ-Proteobacteria and Firmicutes during C227–11 strain infection, on day 1 p.i., when compared to day 0. Therefore, the ỿ-Proteobacteria phylum was predominate among the all analyzed phyla here after day 1p.i., whereas it has reached 70 and 96% on days 2 and 3p.i. respectively (Fig. 2a), with consequent decrease in Firmicutes and Bacteroidetes. Specifically, at the genus level, we observed 44% decrease in *Bacteroides* spp. levels, 49% in *Bifidobacterium* spp. and a small increase of 8% in *Prevotella* spp. on day 1 p.i. when compared to day 0. However, *Lactobacillus* spp. have shown 86% on day 1 and 97% on day 2, reaching higher prevalence on days 2 and 3 p.i. (Fig. 2b). Both WT and C227–11*::qseC* strain were measured in vitro and have similar growth rates (Supplemental Material, Fig. 1S).

Clearly in the absence of the QseC sensor kinase, the C227–11*::qseC* strain infection has shown the predominance of ỿ-Proteobacteria with 82 and 71% on day 1 and day 2 p.i. respectively. However, clear decrease was evidenced, with 8% in ỿ-Proteobacteria, followed by 4% of Bacteroides and an incredibly augment to 88% of Firmicutes, all at day 3 p.i. (Fig. 2c). The genera analysis demonstrated only 3% of *Lactobacillus sp.* on day 1, as well as its gradual increase that reaching 70% on day 2 p.i. and 82% on day 3 p.i., and also showed a low amount of *Prevotella* spp. and *Bacteroides* spp. on day 2 (4 and 8%) and on day 3 (1 and 16%) (Fig. 2d). These microbiota fluctuations QseC-dependent may be related to different chemical signals sensed in the SHIME® model.

Next, the SHIME® model was also employed to verify if the C227–11 interaction with the human intestinal microbiota was enough to trigger Shiga toxin expression, measured via ELISA capture immunoassay from fractions collected in the day 1 and 3 p.i.. The Stx levels were higher in the C227–11 strain infection in both days, with decrease observed in the C227–11*::qseC* strain, respectively in the order of 30 and 40% (Fig. 2e).

### Short-chain fatty acids (SCFA) key production by the human gut microbiota

The microbiota-derived SCFAs have important role during pathogenesis since many enteric pathogens have adapted to distinct SCFA gradients and consequently have evolved mechanisms to regulate virulence gene expression [48, 51, 52].

**Fig. 3:**
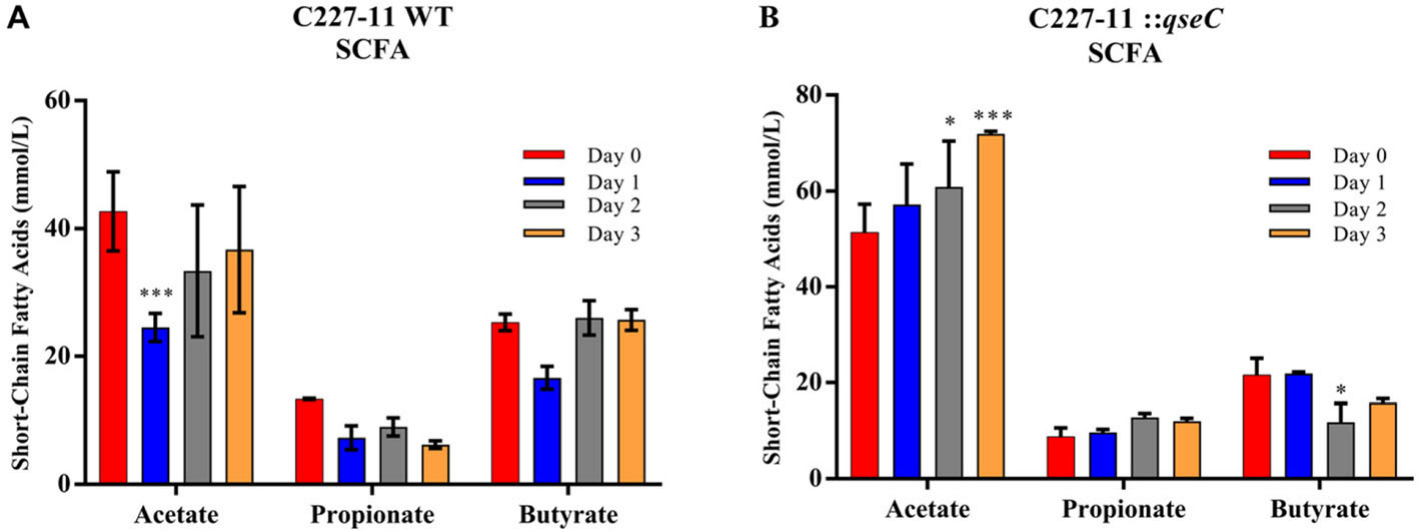
Direct acetate, propionate and butyrate production analysis (mmol/L) from day 0 to day 3.p.i. via gas chromatography. SCFA composition from C227–11 infection period (**a**) (*** *p* = 0.0003) and C227–11::*qseC* (**b**). Analyzes were performed individually for each SCFA compared to day 0. The statistical significance analyzes were performed on GraphPad Prism 7 via one-way ANOVA and Tukey post hoc test (*p = 0.0371, *p = 0.0309, *** *p* = 0.0001)

**Fig. 4:**
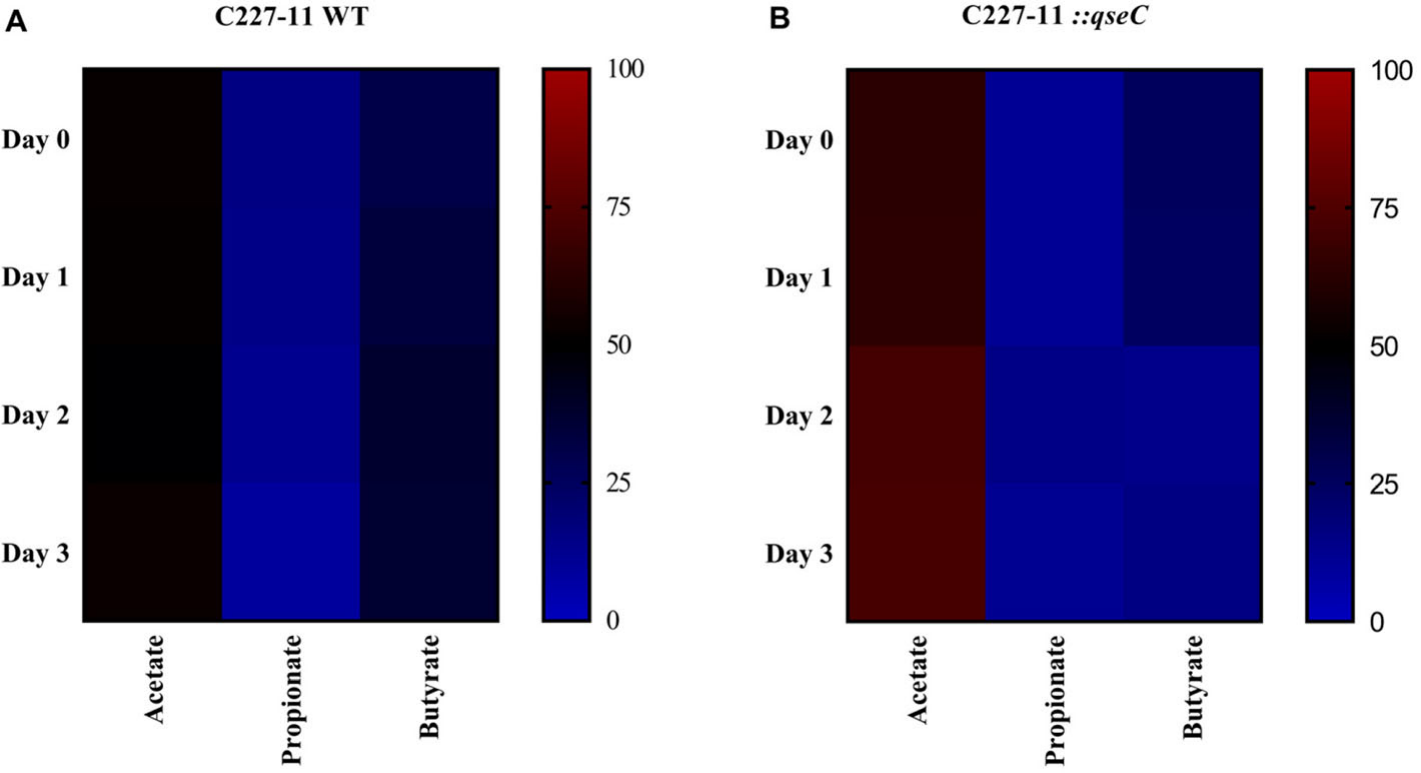
Percentile balance of acetate, propionate and butyrate occurrence from total SCFA production analysis via gas chromatography, daily kinetics during infection, from 0 to day 3 p.i. Differential occurrence in the C227–11(**a**) and C227–11::*qseC* strains (**b**) infection. Gradient from 0% (blue) to 100% (red) concentration of each SCFA

The QseC sensor kinase absence affected directly the microbiota composition to be restored only on day 3 p. i., here we evaluated in the period whether this would also result in differences in the production of the three main SCFAs found in the intestine, acetate, butyrate, propionate (Fig. 3). The SFCA production during SHIME® infection was assessed by measuring it directly via gas chromatography from day 0 to day 3 p.i. The C227–11 strain infection showed a 42.6% decrease in acetate production on day 1 p.i., and no significant difference was observed on the following days, as well as for propionate and butyrate (Figs. 3a and 4a). Gradually, the acetate levels have increased from day 0 to day 3 p.i. during C227–11*::qseC* strain infection, and a distinguished difference of 18.5 and 39.9% was observed, on days 2 and 3p.i., respectively (Fig. 4b). Propionate production has not shown significant changes; conversely, butyrate production has shown a decreased of 45.4% on day 2 p.i., (*p ≤ 0.0371, ***p < 0.0001, *p < 0.0309) (Figs. 3b and 4b). Distinctly, the acetate was more abundant in the two groups evaluated here, and commonly acetate is the more abundant SCFA in the colon [15].

### Interplay between the intestinal microbiota composition and the production of SCFA during the infectious process

The composition and level of SCFA is directly related to the distribution of this gut microbial community [53]. Therefore, we assessed the relationship between the microbiota abundance and the daily percentage of each SCFA. Although changes between phyla and genera were observed during the C227–11 strain experimental period, as seen by the large increase of ỿ-Proteobacteria and *Lactobacillus* spp. on days 2 and 3 p.i. (Fig. 2a and b), and C227–11::*qseC* strain showed correlation between the decrease of ỿ-Proteobacteria, especially after day 2 p.i. (Fig. 2c). In addition to Firmicutes increase on day 3p.i., phylum that includes the *Lactobacillus* genus (Fig. 2c and d). Correspondently to the correlation is observed in bacterial frequency and production of acetate between day 1–3 p.i, increasing both *Lactobacillus* genus and acetate (Fig. 3). The daily proportion among acetate, propionate and butyrate remains steady without significant changes (Fig. 4a), only a gradual increase in acetate production (Fig. 4b).

### QseC sensor kinase also drives the intestinal microbiota shift during C57BL/6 mice infection

**Fig. 5:**
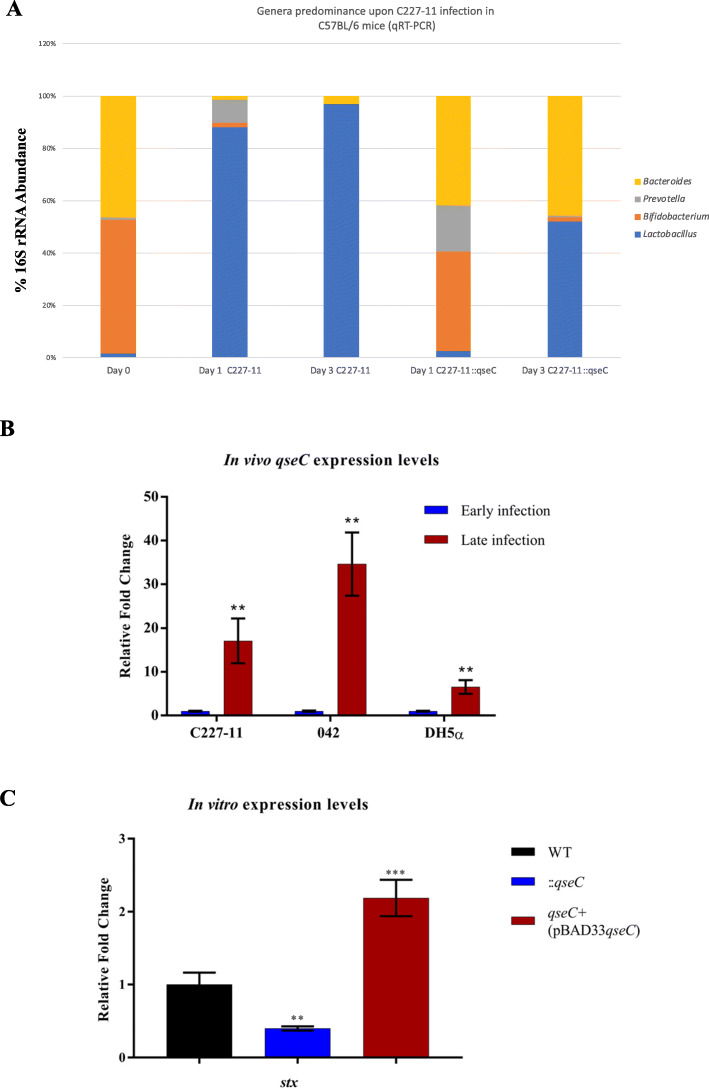
Microbiota predominance during C57BL/6 mice infection, C227–11and C227–11::*qseC* strains (**a**). Expression levels of *qseC* during early and later infection (day 1-3p.i.) of C227–11, 042 and DH5α strains, *p*-values are respectively *p* =0.006 (**), *p* = 0.001 (**) and *p* =0.004 (**) (**b**). Relative expression levels were measured in vitro of *stx2a* gene from the C227–11, C227–11::*qseC*, and C227–11*qseC*^+^ (pBAD33 *qseC*), p =0.01 (**), *p* =0.001 (***) (**c**)

We performed *in vivo* C57BL/6 mice infection to verify and validate the collected data from SHIME® model with a distinct microbiota, together with the virulence features and host response in this model as key components of the mice infection. The genera predominance after infection has shown higher levels of *Lactobacillus* spp. during C227–11 infection in the days 1 and 3 p.i. when compare to C227–11::*qseC* strain (Fig. 5a), where *Lactobacillus* spp. have only increased by day 3 p.i. when compared to *Bacteroides* sp. and *Bifidobacterium* spp. The *qseC* gene expression levels were also higher in strains here tested later during infection of C57BL/6 mice in the day 3 p.i., as the C227–11 and the canonical 042 strain (EAEC pathogenic prototype, O44:H18, Stx^−^), reached 17 and 34-fold change increase, when compared with the control DH5α strain levels in the day 3 p.i., with a lower 6-fold change increase (Fig. 5b), mortality was not observed in the assay employing five (5) mice per group.

The evaluation of the *stx* gene expression levels was also employed to measure an important virulence factor during in vitro LB growth to evaluate the QseC sensor kinase absence impact and its importance to restore the expression levels when the C227–11::*qseC* strain was complemented. The *stx* expression levels are diminished in the C227–11::*qseC* strain as 2-fold lower than WT levels and restored upon *qseC* pBAD33 complementation to similar WT levels (Fig. 5c).

## Discussion

The fundamental role of the intestinal microbiota in resistance to colonization by invading pathogens, which involve both direct and indirect mechanisms, the microbiota together with immune system works as essential lines of host defense. The QseBC 2-component system is employed to sense the environment surroundings and regulate the virulence traits in C227–11 *E.coli* strain and other pathogens, but also to help bacterial colonization and nutrients scavenging [8, 54]. Therefore, it is crucial to understand the intestinal interaction between microbiota and the pathogen during the development of the disease. The QseBC has been shown to be an important way to modulate pathogenic mechanisms of several human enteropathogens, such as EHEC, EAEC*,* and *Salmonella* Typhimurim [34, 55], previously changes in the microbiota abundance driven by QseC and QseE have been described in *Citrobacter rodentium*, a surrogate model for EHEC [56]. Herein, the human colonic intestinal microbiota model was employed to study the chemical signaling influence in the process to better understand the QseC sensor kinase importance in the C227–11 colonization in the SHIME® model and the in vivo model corroborates to drive the microbiota prevalence, that affects the SCFA concentration during infection.

The microbiota imbalance was clear during C227–11 infection in the SHIME® model, upon C227–11 strain infection the *Lactobacillus* spp. and ỿ-Proteobacteria colonization have augmented (Fig. 2a and b). The higher levels of ỿ-Proteobacteria phylum, which includes Gram-negative bacteria, tends to increase intestinal permeability and trigger the inflammatory process by releasing LPS (lipopolysaccharide), as a bacterial endotoxin. This phylum includes very critical enteropathogens such *as E. coli* pathotypes, *Salmonella*, *Yersinia*, *Vibrio* and *Pseudomonas*, thus, its increase may be permissive to human health [57–59]. The absence of QseC sensor kinase within the SHIME® model during microbiota interaction seems to attenuate the C227–11 strain upon microbiota interaction, with lower secretion of Shiga toxin noted in the C227–11*::qseC* than WT strain in the ELISA immunoassay direct assayed from the intestinal extracts (Fig. 2e), the direct link between QseC and Shiga toxin in the C227–11 strain demands further studies. However, the data here raises the point about Shiga toxin possible implication in C227–11 strain during the human intestinal microbiota competition, since the model lacks the presence of host tissue.

The QseC sensor seems to be key player during infection, whereas the C227–11*::qseC* strain infection in the SHIME® model had delayed recovery of the indigenous microbiota only by day 3 p.i. (Fig. 2c and d). It was also observed high levels of Firmicutes (Fig. 2c), consider as the main bacteriocins producer [60]. Higher abundance of *Bacteroides* spp. and *Bifidobacterium* spp. (Fig. 2d) was observed after C227–11*::qseC* strain infection, both important bacteria to increase the acetate levels [51, 61]. The C227–11*::qseC* mutant infection has presented a distinct balance of microbiota composition in the SHIME model, this difference is more prominent in the 2 initial days, by day 3 p.i. the differences in composition became less evident. These data imply QseC major role in the initial infection in the SHIME® model. Again, the indigenous intestinal microbiota shows its importance during maintenance of gastrointestinal homeostasis and the host’s intestinal health [62]. Commensal and probiotic intestinal bacteria are important against enteric disease by several mechanisms, including competitive exclusion, adhesion, and production of antimicrobial compounds [63]. There are limitations for in vivo EAEC infection models, since the colonization of these bacteria need a partial microbiota depletion, affecting the intestinal conditions, additionally most of the animal models do not reproduce the diarrheagenic component of the enteropathogens disease. Therefore, the colonic model SHIME® may be employed as important possibility to further investigate the interaction between the pathogen and the human microbiota in conditions similar to intestine in a controlled environment.

Members of the Bacteroidetes phylum, more specifically *Bacteroides* spp., are primarily responsible for the acetate and propionate production, while members of the Firmicutes phylum, are the main producers of butyrate [64]. However, it is known that members of the four main phyla contribute substantially to the production of SCFA, thus, the imbalance of the intestinal microbiota may result in changes in the production of these metabolites and health disorders [52, 65].

Recently, we have shown that QseBC system signaling pathway interruption has substantially reduced the EAEC colonization and virulence gene expression in mice model of infection of *fimH*, that encodes de Type I fimbriae pilin and QseC essential role during the in vivo model to modulate the microbiota imbalance at Phyla level in mice during EAEC infection [42]. Nonetheless, similar studies have evidenced QseC importance during the *Citrobacter rodentium* infection, a natural pathogen of mice that mimics EHEC infection in vivo, the reduction of colonization and virulence factors expression such as *espA* and *tir*, in addition to decrease the ỿ-Proteobacteria levels [56]. The data presented here indicate how the pathogens such as C227–11 may play a role in the intestinal microbiota balance, therefore contribute to microbiota composition and abundance of distinct intestinal bacteria and metabolites as direct effect caused by the pathogen presence (Figs. 2, 3 and 4).

The SCFA adequate levels in the intestine are important for human health, because they perform vital functions for the gastrointestinal system [66]. In additional, the SCFAs have an important role during EHEC pathogenesis, because they impact EHEC gene regulation. Butyrate works as a signal in EHEC by enhancing the expression of the T3SS and flagellar genes [67]. Here, the C227–11 strain infection have shown significant change in the SCFA levels, such as 42% decrease in acetate production on day 1 p.i. (Fig. 3a). Probably, the QseC histidine kinase sensor absence in the C227–11 mutant strain contributed to a positive microbiota modulation during infection period, and to increased acetate production (Fig. 3b).

Previously work with these metabolites have also shown that they may cross the bacterial membrane and accumulate in the cytoplasm, leading to the influx of protons and consequent intracellular acidification [52].. Higher levels of acetate produced by *Bifidobacteria* spp. were correlated with protection against EHEC O157:H7 infection in mice. Moreover, acetate prevented the decreases in transepithelial electrical resistance, which contributes to lower translocation of Shiga toxin into the bloodstream [68], conversely the translocation is helped by AggR-regulated AAF/I adherence to intestinal epithelium enhancing inflammation [19–24]. Acid conditions in vitro inhibited *S. dublin* and EHEC O157:H7 growth in the presence of SCFA, such as acetate [69, 70]. The levels of acetate, propionate and butyrate in the intestinal lumen usually sustains a ratio of approximately 60:20:20 mM, and their abundance is related to the microbiota composition, diet, host genetic features and intestinal trafficking [71, 72]. For instance, during *Shigella flexneri* infection in vivo, another important human intestinal pathogen, the administration of SCFA, such as acetate, propionate and butyrate, in distinct 60:30:40 mM proportion, resulted in decreased colonization and improved clinical symptoms [73]. The intestinal acetate is mostly produced by bacteria present in the colon, such as *Lactobacillus* spp., *Bifidobacterium* spp. and *Bacteroides* spp. [74], thus, the decline of this SCFA on day 1 p.i. during the C227–11 strain infection (Fig. 3a) is probably related to the sudden drop of these species, leading to an imbalance of its production in this period and reestablishment in the following days (Fig. 2a). On the other hand, the proportion between acetate, propionate and butyrate in each day, remained without significant changes, and could not be directly correlated with microbiota shift in the evaluate period (Figs. 2a, b and 4a). Furthermore, the increase in acetate production on days 2 and 3 p.i. in the C227–11::*qseC* (Figs. 3b and 4b) are directly correlated with high levels of *Lactobacillus* spp., *Bacteroides* spp., and the Firmicutes phylum (Fig. 2c and d), whereas Firmicutes are well known to establish them as the main producer of butyrate [75]. Together with SHIME data, herein the performed in vivo mice infection model for 042 and C227–11 strains with partial disrupted microbiota [44]. These assays have validated that C227–11::*qseC* infection in vivo also presented lower colonization levels of *Lactobacillus* spp. after day 1 p.i.. than the WT strain (Fig. 5a). During C227–11 infection the higher levels of *Lactobacillus* spp. may be correlated with the acetate levels, in the SHIME infection they increase as the progression of infection by day 3 p.i., again specially in the C227–11::*qseC* strain. Clearly, the QseC seems to be important during the progression of infection, as observed in vivo by the *qseC* gene expression levels in both EAEC strains (Fig. 5b). Virulence factors such as *stx* gene expression levels were assayed in vitro during LB growth to evaluate the QseC absence as important factor to attenuate the C227–11 strain (Fig. 5c), like also observed in the C227–11::*qseC* strain in SHIME® and C57BL/6 infection. The C227–11 has multiple virulence factors probably triggered during different points of the infection in the mice model [42]. However, Shiga toxin important in the host inflammation, may also be employed to interact with the intestinal microbiota, as it is increased in the SHIME® model (Fig. 2e). The microbiota competition and interaction with enteropathogens may be changeling and very fascinating.

## Conclusions

The dynamic view of the microbiome, microbial metabolites and infectious process by Shiga toxin-producing C227–11 provided novel insights into the interplay of pathogenic bacteria infection with the microbiota. The QseC kinase sensor seems to modulate the human intestinal microbiota shift during the infectious process by Shiga toxin-producing EAEC C227–11. The SHIME® infection model has proven to be an efficient alternative tool, and correlated with in vivo data, to study pathogen and human microbiota interactions. The QseC sensor kinase of C227–11 strain helped to remodel the gut microbiota, driving distinct abundance in microbiota composition and changed SCFA levels. Finally, our results emphasize the QseC potential as a target for studies in the development of new therapies for EAEC infections.

## Methods

### Strains

In this study, we used the EAEC O104:H4 C227–11 strain isolated from elderly patient with hemorrhagic colitis, hospitalized during the outbreak period [29]. The C227–11::*qseC* strain, gene that encodes the QseC kinase sensor, was previously constructed via pJP5603 suicide vector [42], and all other strains used are listed (Table 1). The *qseC* mutant strain (::*qseC*) was complemented with the *qseC* gene cloned into pBAD33 (SacI/KpnI) as constitutive active vector as previously described [42]. The strains were grown in Luria-Bertani medium (Invitrogen™) with 100 μg/mL of streptomycin, under agitation at 37 °C overnight (16-18 h).

**Table 1:** Strains used in this study

Strain	Main Features	Reference
C227–11	wild type, pAA, AggR, AAF/I, Stx2a, Pic, SigA, SepA	[29]
C227–11::*qseC*	*qseC* mutant,inserted with R6K-based suicide vector, Km^r^	[42]
C227–11 *qseC*+	*qseC* complemented strain with constitutive low-copy pBAD33- Gm^r^	[42]
DH5α	*E. coli supE44* Δ*lacU169*(ϕ80*lac*ZΔM15) *hsdR17*	Stratagene
042	EAEC prototype strain, O44:H18 (Stx^−^) (isolated in diarrheal case in Peru)	[76]

### SHIME® model set up

The Simulator of Human Intestinal Microbial Ecosystem (SHIME®) was used to simulated the human digestion process. The SHIME® reactor is computer-controlled and consists of 5 closed compartments representing the stomach, small intestine, ascending colon, transverse colon and descending colon [46]. The reactor was adapted for this study, where the transverse and descending colon were replaced by the triplicate of the ascending colon (pH 5.6–5.9), aiming to obtain replicates of the experiment for statistical comparison of the data (Fig. 1a), with experimental settings as previously described [49].

### Microbiota colonization

The compartments were colonized with feces earlier collected and stored from three healthy volunteers with ages between 18 and 22 years old, according to the procedures [77], adapted to this study [78], and sampling prepared as previously described [49]. All feeding components (Sigma Aldrich, USA) are listed in Table 2.

**Table 2:** Feed and pancreatic juice composition used in the SHIME®

Feed Component^a^	g/L
Arabinogalactan	1.0
Pectin	2.0
Xylan	1.0
Potato starch	3.0
Glucose	0.4
Yeast extract	3.0
Peptone	1.0
Mucin	4.0
Cystein	0.5
Sterile distilled water	q.s.p^b^
**Pancreatic juice**	
Oxgall	6
NaHCO3	12.5
Pancreatin	0.9
Sterile distilled water	q.s.p

^a^ Sigma-Aldrich

^b^*Quantum Satis para* (for Liter)

### SHIME® infection

The experimental period in the SHIME® was performed continuously during 5 weeks, as illustrated in Fig. 1b. In the microbiota stabilization period (control), the feed medium (240 mL) and pancreatic juice (60 mL) were inserted into the system as previously described for 14 days [79, 80] (Table 2). A 2 weeks period of stabilization was performed and the first treatment was administered for 72 h. The first infection consisted of 10^10^ CFU/mL of *E. coli* C227–11 strain, 240 mL of feed medium and pancreatic juice (60 mL) were added. Between the treatments, a 72 h washout period was performed [49]. After the washout, under similar conditions during the second infection (72 h) started with the 10^10^ CFU/mL *E. coli* C227–11::*qseC* strain in the same conditions, all the experiments were performed in biological triplicates. Selective medium was used to isolate Gram-negative bacilli based on lactose fermentation from the samples of the reactors, when necessary. The EAEC presence and absence was checked in the washout via PCR of exclusive C227–11 gene (Table 3).

**Table 3:** Oligonucleotides sequences used in this study

Target	Primer sequence (5′-3′)	Reference
**q-RT-PCR**		
Bacteroidetes	Forward – CRAACAGGATTAGATACCCT Reverse – GGTAAGGTTCCTCGCGTAT	[81]
Firmicutes	Forward – TGAAACTYAAAGGAATTGACG Reverse – ACCATGCACCACCTGTC	[82]
ỿ-Proteobacteria	Forward - TCGTCAGCTCGTGTYGTGA Reverse – CGTAAGGGCCATGATG	[82]
Eubacteria	Forward – ACTCCTACGGGAGGCAGCAGT Reverse - ATTACCGCGGCTGCTGGC	[83]
*Bacteroides* spp.	Forward – CGATGGATAGGGGTTCTGAGAGGA Reverse - GCTGGCACGGAGTTAGCCGA	[50]
*Prevotella* spp.	Forward – CACCAAGGCGACGATCA Reverse - GGATAACGCCYGGACCT	[50]
*Bifidobacterium* spp.	Forward- TCGCGTC(C/T)GGTGTGAAAG Reverse - CCACATCCAGC(A/G)TCCAC’	[84]
*Lactobacillus* spp.	Forward - AGCAGTAGGGAATCTTCCA Reverse – CACCGCTACACATGGAG	[84]
*stx2a*	Forward – ACCCCACCGGGCAGTT Reverse - GGTCAAAACGCGCCTGATA	[39]
*rpoA*	Forward – GCGCTCATCTTCTTCCGAAT Reverse - CGCGGTCGTGGTTATGTG	[55]
**PCR**		
*stx2a*	Forward – CAGTCGTCACTCACTGGTTTCATCA Reverse - GGATATTCTCCCCACTCTGACACC	[85]

### Analysis of short-chain fatty acids (SCFA)

The short chain fatty acids were analyzed via gas chromatography as previously described with minor modifications [77]. The samples (*n* = 3, second week of the colon reactors) were centrifuged (14,000 x g, 5 min) and 2 mL of the supernatant stored for analysis. Analytical curves were constructed from stock solutions of the acids of interest (acetic, propionic and butyric). The samples were filtered through Millex® filters (0.45 μm) into flasks and then injected into an Agilent HP-6890 gas chromatograph equipped with an Agilent model HP-5975 mass-selective detector. A DB-WAX capillary column (60 m × 0.25 mm × 0.25 μm) was used under the following conditions: Injector temperature = 220 °C, column = 35 °C, 2 °C/minute, 38 °C; 10 °C/minute, 75 °C; 35 °C/minute, 120 °C (1 min); 10 °C / min, 170 °C (2 min); 40 °C / minute, 170 °C (2 min), and detector = 250 °C. Helium was used as the carrier gas at a flow rate of 1 mL/minute.

### RNA extraction

Samples were collected individually from each ascending colon reactor from day 0 to day 3 p.i. To disrupt the cells and preserve the genetic material, 1 mL of TRIzol® (Ambion) was used for each 100 mg of fecal content. To analyze virulence genes expression, RNA was extracted from late exponential growth phase in LB (O.D._600_ 1.0). The RNA was purified by RiboPure™ Bacteria Kit (Ambion), according to manufacturer instructions.

### Microbiota abundance and gene expression analysis via qRT-PCR

The relative microbiota abundance and relative quantification of gene expression were analyzed via qRT-PCR (Real-Time Quantitative Reverse Transcription PCR), and the reactions was performed using the QuantStudio™ 3 Real-Time PCR Systems (Thermo Fisher Scientific). The reactions were performed in triplicates and final volume of 20 μL, containing Master Mix SYBR®, Multi-scribe® Reverse Transcriptase, RNAse inhibitor (Thermo Fisher Scientific) and 100 ng of RNA. The primers used to analyze phyla, genera, endogenous controls, and individual genes are listed in Table 3. The samples collected independently of each reactor in SHIME® model in the time period listed. As endogenous control, was employed *16S rRNA* (Domain Bacteria) for the total of bacteria present in each reactor or *rpoA* (RNA polymerase subunit A) to gene expression levels. The data were analyzed via Comparative critical threshold (ΔΔCT) [86].

### SHIME® washout step controlled via PCR

The PCR (Polymerase Chain Reaction) reactions to verify the absence of O104:H4 C227–11 or C227–11*::qseC* strains in the washout period, were performed from samples collected independently of each reactor, and lized at 100 °C for 5 min in heat block. This output was employed as DNA template and *stx2a* set of primers for in the reaction (Table 3). All DNA amplification reactions were performed in the T100™ Thermal Cycler (Bio Rad), with an annealing temperature of 55 °C and extension at 72 °C. The 282 bp PCR product was analyzed on a 1% agarose gel and the images captured on the ChemiDoc MP Imaging System® (Bio-Rad). Conditions for each 50 uL reaction: 35.3 uL of nuclease-free water, 5 uL of buffer (1x), 1uL forward prime (0.2 μM), 1uL of reverse primer (0.2uM), 1.5 μL MgCl_2_ (1.5 mM), 1uL of DNTP mix (0.2 mM each), 5uL of DNA template from lized cells, 0.2 uL of Invitrogen™ Platinum™ Taq DNA Polymerase (1UI).

### Capture ELISA immunoassay

Microtiter plates (C96 Polysorp - NUNC) were incubated at 37 °C for 2 h and then further 4 °C for 16 h with 25 μg/mL of Stx2 polyclonal antibody (pAb) in carbonate-bicarbonate-buffered, pH 9.6. BSA 1% was added as blocking and incubated for 1 h at 37 °C. Material was collected from the reactors and they were incubated for 1 h at 37 °C. Toxin bound to Stx2 pAb were then detected with 5 μg/mL of Stx2 monoclonal antibody followed by anti-mouse IgG peroxidase (Sigma, 15,000) and then with 10 mg/plate of OPD in the presence of hydrogen peroxide. Between incubations the plates were washed three times with PBS-tween 0,05%. Normal microbiota and purified toxin stx2 were used as control. All experiments were carried out in duplicate and results correspond to three independent experiments.

### In vivo microbiota assays

The employed mice were acquired from CEMIB/UNICAMP and maintained at our Animal Facility in the Biological Sciences Department at School of Pharmaceutical Sciences/UNESP. The experiment with animals was previously approved by the Animal Ethics Committee (CEUA/FCF/Car 23/2016). Animals were 3- to 5-week-old female C57BL/6 J UNIB mice, weighing between 12 and 15 g. They were pretreated with 20 mg/kg of ampicillin via oral gavage, 24 h before infection, to allow better colonization. Assays were divided into four experimental groups of animals with five (5) mice per group; one group was inoculated with *E. coli* K-12 DH5a (non-pathogenic) as a negative control. The other three groups were infected, respectively, with C227–11, C227–11::*qseC,* and EAEC 042 strains. All experiments were repeated at least twice to ensure the results presented here. Strains were cultivated for 16 to 18 h, centrifuged, and resuspended in PBS. Animals were infected with 10^10^ bacteria via oral gavage. Feces were collected from mice from days 1 to 3 p.i. to be recover, weight loss was monitored and considered for animals with a decrease of at least 5% of total body mass and gene expression was determinated by qRT-PCR as previously described [42].

### Statistical analysis

The data were analyzed in the GraphPad Prism 7, and the statistical significance among the groups was determined using the Oneway analysis of variance (ANOVA). *P* values ≤0.05 were considered statistically significant. The ELISA immunoassay statistical significance analyzes were performed via t-test.

## Supplementary Information

**Additional file 1.**

## Data Availability

All data generated or analyzed during this study are included in this published article.
