# Correction to: Human microbiota modulation via QseC sensor kinase mediated in the *Escherichia coli* O104:H4 outbreak strain infection in microbiome model

**DOI:** 10.1186/s12866-021-02266-3

**Published:** 2021-08-24

**Authors:** Tamara Renata Machado Ribeiro, Mateus Kawata Salgaço, Maria Angela Tallarico Adorno, Miriam Aparecida da Silva, Roxane Maria Fontes Piazza, Katia Sivieri, Cristiano Gallina Moreira

**Affiliations:** 1 Department of Biological Sciences, School of Pharmaceutical Sciences, São Paulo State University (UNESP), Araraquara, SP Brazil; 2 Department of Food and Nutrition, School of Pharmaceutical Sciences, São Paulo State University, (UNESP), Araraquara, SP Brazil; 3 Department of Hydraulics and Sanitation, School of Engineering of São Carlos, University of São Paulo (USP), São Carlos, SP Brazil; 4 Bacteriology Laboratoty, Butantan Institute, São Paulo, SP Brazil

**Correction to: BMC Microbiology (2021) 21:163**

**https://doi.org/10.1186/s12866-021-02220-3**

Following the publication of the original article [1], we were notified that the captions for Figs. 2, 3 and 5 needed adjustments.

Original captions:

- Fig. 2: “Microbiota predominance modulated via QseC during C227–11 infection in the SHIME® model. Relative microbiota abundance analysis via qRT-PCR of 16 s rRNA of phyla and genera. Microbiota composition from days 0 to 3 p.i with strain C227–11 infection, respectively, phyla and genera (a and b), and with strain C227–11::qseC infection, respectively, phyla and genera (c and d). ELISA Immunoassay capture to measure the Stx levels from the output collected during the SHIME® infection, day 1, ** p = 0.002 and 3 p.i., ** p = 0.009 (e). The statistical significance analyzes were performed on GraphPad Prism 7 via t-test”
- Fig. 3: “Direct acetate, propionate and butyrate production analysis (mmol/L) from day 0 to day 3.p.i. via gas chromatography. SCFA composition from C227–11 infection period (a) (*** p = 0.0003) and C227–11::qseC (b). Analyzes were performed individually for each SCFA compared to day 0. The statistical significance analyzes were performed on GraphPad Prism 7 via one-way ANOVA and Tukey post hoc test (*p = 0.0371, *p = 0.0309, *** p = 0.0001)”
- Fig. 5: “Microbiota predominance during C57BL/6 mice infection, C227–11and C227–11::qseC strains (a). Expression levels of qseC during early and later infection (day 1-3p.i.) of C227–11, 042 and DH5α strains, p-values are respectively p =0.006 (**), p = 0.001 (**) and p = 0.004 (**) (b). Relative expression levels were measured in vitro of stx2a gene from the C227–11, C227–11::qseC, and C227–11qseC+ (pBAD33 qseC), p = 0.01 (**), p = 0.001 (***) (c)”

Corrected captions:

- Fig. 2: “Microbiota predominance modulated via QseC during C227–11 infection in the SHIME® model. Relative microbiota abundance analysis via qRT-PCR of 16 s rRNA of phyla and genera. Microbiota composition from days 0 to 3 p.i with strain C227–11 infection, respectively, phyla and genera (a and b), and with strain C227– 11::qseC infection, respectively, phyla and genera (c and d). ELISA Immunoassay capture to measure the Stx levels from the output collected during the SHIME® infection, day 1, ** p = 0.002 and 3 p.i., ** p = 0.009 (e). The statistical significance analyzes were performed on GraphPad Prism 7 via t-test”
- Fig. 3: “Direct acetate, propionate and butyrate production analysis (mmol/L) from day 0 to day 3.p.i. via gas chromatography. SCFA composition from C227–11 infection period (a) (*** p = 0.0003) and C227–11::qseC (b). Analyzes were performed individually for each SCFA compared to day 0. The statistical significance analyzes were performed on GraphPad Prism 7 via one-way ANOVA and Tukey post hoc test (*p = 0.0371, *p = 0.0309, *** p = 0.0001)”
- “Fig. 5: Microbiota predominance during C57BL/6 mice infection, C227–11and C227–11::qseC strains (a). Expression levels of qseC during early and later infection (day 1-3p.i.) of C227–11, 042 and DH5α strains, p-values are respectively p =0.006 (**), p = 0.001 (**) and p = 0.004 (**) (b). Relative expression levels were measured in vitro of stx2a gene from the C227–11, C227–11::qseC, and C227–11qseC+ (pBAD33 qseC), p = 0.01 (**), p = 0.001 (***) (c)”

The original article has been corrected.
