# Supplementary figures and images for: Human microbiota modulation via QseC sensor kinase mediated in the Escherichia coli O104:H4 outbreak strain infection in microbiome model
Source: BMC Microbiol. 2021 Jun 2;21:163. doi: 10.1186/s12866-021-02220-3 (PMC8170955; doi:10.1186/s12866-021-02220-3)

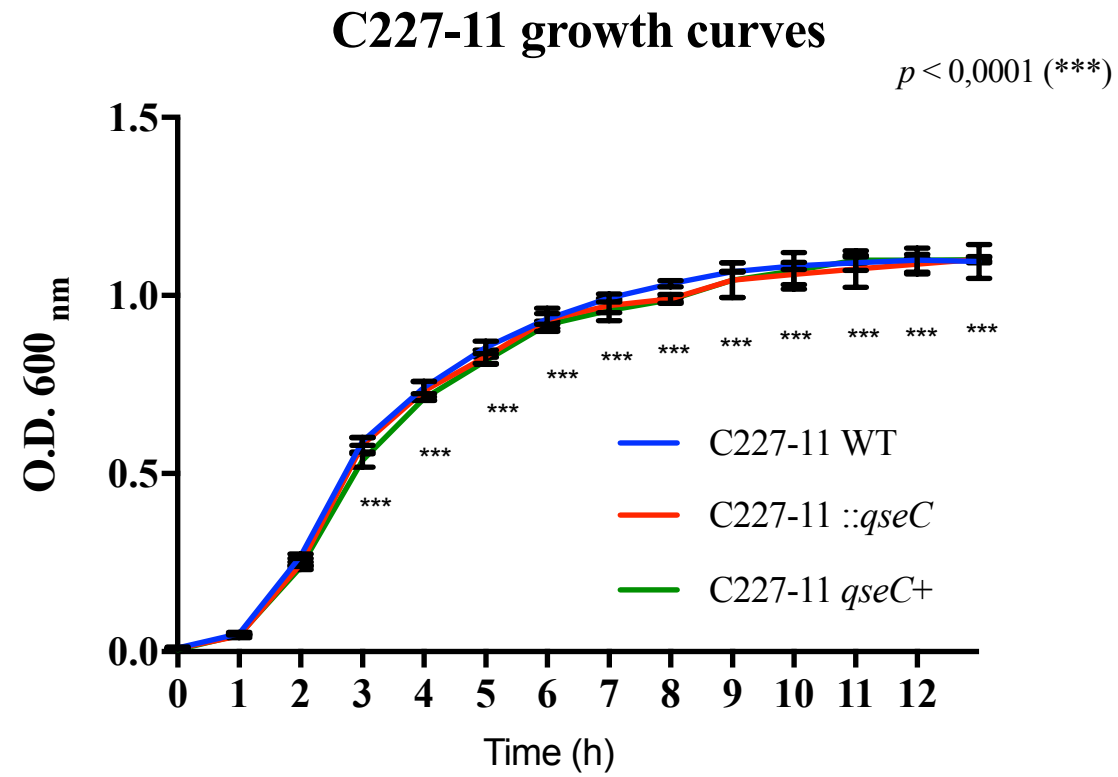

**Sup. Mat. 1** Growth curve of the E. coli C227-11 strain in LB media

Supplement: Supplementary file 1 — Additional file 1. [file 12866_2021_2220_MOESM1_ESM.pdf]
